# Use of Muller's maneuver in the evaluation of patients with sleep apnea - literature review

**DOI:** 10.1016/S1808-8694(15)30667-4

**Published:** 2015-10-19

**Authors:** Maria Claudia Mattos Soares, Ana Carolina Raposo Sallum, Michele Themis Moraes Gonçalves, Fernanda Louise Martinho Haddad, Luís Carlos Gregório

**Affiliations:** 1 MSc. In Otorhinolaryngology - UNIFESP-EPM; 2 MD. Resident Physician in Otorhinolaryngology - UNIFESP-EPM; 3 MD. Resident Physician in Otorhinolaryngology - UNIFESP-EPM; 4 MSc in Otorhinolaryngology - UNIFESP-EPM; 5 PhD in Otorhinolaryngology - UNIFESP-EPM, Head of the Otolaryngology and Head and Neck Surgery Program - UNIFESP-EPM

**Keywords:** sleep apnea, endoscopy, obstructive

## Abstract

Sleep apnea-hypopnea syndrome was described twenty years ago, and since then there have been doubts and controversies regarding it. Fiberoptic nasopharyngoscopy with Muller's maneuver, first described by Borowieck and Sassin (1983), is among them.

**Aim:**

Careful literature review on Muller's maneuver, regarding whether it can predict the sucess of uvulopalatopharyngoplasty, location of upper airway obstruction and severity of the disorder.

**Discussion and literature rewiew:**

Literature has shown that there isn't a consensus about the use of Muller's maneuver. In spite of being technically easy, inexpensive and widely used, it is very unespecific and subjective.

**Conclusion:**

The importance of Muller's maneuver in evaluating apneic patients has been questioned, because there are controversies whether it can predict the sucess of uvulopalatopharyngoplasty, location of upper airway obstruction and severity of the disease.

## INTRODUCTION

Studies involving the main characteristics of patients with Obstructive Sleep Apnea Syndrome (OSAS) started with Burwell, Robin, Waley and Bickelmann (1956)^1^. They described the Pickwickian Syndrome, in homage to the English writer Charles Dickens, author of the classic “The Posthumous Papers of the Pickwickian Club” (1837), which main character was an obese, sleepy and snoring boy. The syndrome is classically made up of obesity, hypercapnia, cor pulmonale, erithrocytosis and excessive day-time sleepiness.

Obesity causes overload and consequent respiratory depression, leading to hypercapnia and hypoxemia; such blood gas unbalance could explain day-time excessive sleepiness^2^,^3^.

During the 60's, when polysomnography (PSG) became available, European authors started to investigate the Pickwickian syndrome as a sleep respiratory disorder and concluded that day-time excessive sleepiness came from sleep fragmentation, and not from changes in blood gases^3^,^4^.

In Italy, 1972, the first sleep-related symposium on respiratory disorders was held, when Guilleminault et al.^5^ established the terminology: Obstructive Sleep Apnea Syndrome (OSAS), characterized by excessive day-time sleepiness and apnea episodes detected by PSG. The concept of hypopnea was first described in 1979 as superficial breathing causing denaturation during sleep^6^. It was almost a decade later, in 1988, that the term OSA was established^7^.

According to the American Academy of Sleep Disorders, in 2005^8^, the proper terminology is OSAS, since there is no evidence that the physiopathology and the clinical repercussions of the apnea and hypopnea events are different.

Numerous theories have been proposed to explain the disease pathophysiology, which is multifactorial, partially stemming from anatomical variations of the upper airways and the facial skeleton associated with neuromuscular alterations in the pharynx^9^,^10^. Despite our knowledge that apneic individuals have pharynxes more prone to colapse^11^, ^12^, ^13^, we still have a great difficulty in assessing exactly which point in the pharynx suffers the worst collapse during sleep. That happens because the evaluations are all carried out with the patient awake, when his/her muscle tone is maintained and during sleep there is a progressive hypotonia, reaching full atony during the REM (“rapid eye movement”) sleep.

Since the upper airway (UAW) is the OSA obstruction site and the working ground of otorhinolaryngologists, it has been carefully studied by specialists of the field. In these regards, the Nasofibroscopy-Assisted Muller's Maneuver (NMM) through Nasofibroscopy, described in 1983^14^, is the scope of the present paper.

## GOAL

Literature review, with a critical and comparative analysis regarding the NMM's capacity to detect the upper airway point of collapse, to predict the success of uvulopalatopharyngoplasty (UPPP) and the OSA severity.

## METHOD

Asystematic review. We studied papers indexed in the LILACS and MEDLINE databases from 1950 all the way to 2007.

## LITERATURE REVIEW AND DISCUSSION

In 1964 the first study was published involving oropharynx characteristics in snoring adults, showed that 91% of these patients had a narrow pharynx, elongated soft palate and uvula^15^, in which they proposed the mucosal resection of the anterior tonsillar pillar and part of the uvula, with satisfactory results.

These ideas were refined at the same time that studies started to confirm the hypothesis that the oropharynx was an important OAS obstruction site^16^. Based on these facts, in 1981^17^ the UPPP was described, with a 50% success rate, a true landmark in the treatment of this disorder. At this time, the UPPP became quite popular, being the most frequently performed procedure for the treatment of snoring and sleep apnea during the 80's and 90's^18^. Since then, otolaryngologists took on a very important role in OSA diagnosis and treatment.

Nonetheless, surgical results were better in patients with snoring and only 50% of the apneic patients benefited from the surgery – 50% of HAI (hypopnea Apnea Index) reduction was considered a success^19^.

In later studies, when the success rate of 50% reduction in HAI was used, associated with a HAI below ^20^, the success rate found was 40%^20^. Numerous factors were held responsible for these poor results, such as disease severity, multiple obstruction sites, obesity and anatomical alterations on the mandible and maxilla^20^. The success of UPPP varies between 8 and 100% in the literature; however, there are numerous techniques and success criteria used, which make it very difficult to compare the data^21^, ^22^, ^23^, ^24^, ^25^.

Even then, UPPP remains as the most frequently performed surgical procedure for the treatment of OAS^20^,^25^, ^26^, ^27^, ^28^.

With the intent of properly selecting the apneic patients that could be successfully treated by UPPP, determining a trend towards pharyngeal collapse and laxity, in 1983^14^ the NMM was described, in which the patient undergoes a forced inhaling effort, having both the nose and mouth closed and the examiner, with the nasofibroscope located in the retrolingual region, observes the side-to-side and antero-posterior narrowing of the pharyngeal walls; the maneuver is repeated with the device in the retropalatal region. The patients with retropalatal collapse would be the best candidates to UPPP^29^,^30^.

The capacity of NMM in forecasting UPPP success was assessed in a group of apneic patients^31^, for which 30 patients with retropalatal collapse were studied through the NMM; of those, 22 (73%) had a minimum reduction of 50% in the hypopnea apnea index (HAI) in relation to baseline, considered a surgical success by the author; notwithstanding, in the literature the most accepted criterion is a minimum reduction of 50% in the HAI and HAI<2018. The study was carried out in apneic patients only and surgery was offered only to those patients with retropalatal obstruction; thus the lack of a control group, as well as the success criterion used, limits a proper analysis of the results presented.

The same study was later on reproduced^32^ in 24 patients with moderate or severe OSA, and surgery was performed in patients with retropalatal (15) and retrolingual (9) collapse. Success rate, using the same criteria aforementioned, in the first group was 33.3% (5) and that of failure in the second group was 77.7% (7). This study suggests that the NMM is not capable of predicting those that would have a good surgical result; however, it can select those that would fail.

A retrospective study was carried out in 30 patients with OSAS^33^ and the NMM predictive value together with that of cephalometry were compared in the selection of patients for UPPP. NMM was not able to forecast those patients that would be successfully treated by UPPP (50% reduction in HAI and HAI< 20) or surgical failure. The authors propose a model grouping up three cephalometric measures (distance between the hyoid and the mandibular plane, skull-neck angle and length of the maxilla) and hypersomnia, which is able to properly select these patients in 83% of the cases.

A similar study to the one aforementioned, involving 53 apneic patients^34^ considered an HAI reduction of 50% as surgical success. Both NMM and cephalometry were capable of selecting successful or unsuccessful patients for UPPP. The maneuver was carried out with the patient laying down and seating up, and there was no difference between them.

The studies aforementioned show that NMM alone is not a good method to detect the pharynx collapse site and predict UPPP success, since patients with retropalatal narrowing seen at NMM had surgical results short of what was expected.

In 1999, Ritter et al.^35^ published the only study in the literature with a quantitative analysis of NMM, using software to measure the area and the diameters of the retropalatal and retroglossal areas in the baseline and during the maneuver, and also a pressure transducer to control the patient's inspiratory effort. Thus, the authors excluded a major criticism to this procedure: patient and examiner's subjectiveness. The test was carried out in normal patients, laying down and in a seating position. In the retropalatal region, there was a reduction in the area and the side-to-side diameter during Muller's maneuver when compared to baseline values. The retrolingual region did not show any significant area reduction, because besides the side-to-side narrowing, there was an antero-posterior diameter enlargement. In both, there was no difference between NMM laying down or seating up.

In a OSAS consensus held in 2002, Rombaux et al. ^36^ suggest that NMM should be performed with a pressure transducer under a −20 cm H_2_O inspiratory effort to minimize patient's subjectiveness to the test; however, they say it would not be feasible in daily practice. Nonetheless, its indication is maintained in the consensus because it is easy to perform, and it is time and cost effective.

Many patients are incapable of producing the proper inspiratory effort, and there may be differences among the patients and even different results from the same patient in different tests. Any test based on the patient's collaboration has some level of variability^37^.

The maneuver's reproducibility was studied38 between two different examiners, one of them a first year resident and the other was a professor in the institution and there was a good correlation between them. Nonetheless, it is a renowned institution (Stanford University) to treat sleep disorders, with a large number of apneic patients and physicians used to and trained to assess them^38^.

By the same token, the agreement between the two examiners during NMM was also assessed^39^. Insofar as the pharyngeal narrowing is concerned, there was an agreement in 27 of 42 tests and disagreement in 15. In the retropalatal region, there was a good agreement (16 en 21 tests), with kappa index = 0.63. The results showed a weak agreement in the retroglossal region (11 in 21 tests), with a kappa index = 0.3.

Therefore, there is a tendency in the literature of stating that the NMM reproducibility by examiners is challengeable.

Studying the correlation with OSAS severity, one can conclude that the Muller's maneuver is not a good disease severity predictor40. Other authors found a moderate agreement (72%)^38^.

According to the literature, NMM advantages are: easy to do for patient and examiner, time and cost effective and broadly divulged^36^,^41^. The disadvantaged are: invasive, subjective for the patient and examiner, questionable reproducibility, possible dynamic modification of the upper airway dynamics because of the endoscope present, which does not necessarily reproduces the apnea episode^32^,^37^,^41^.

## FINAL REMARKS

Muller's maneuver is a tool used to help the otolaryngologist in handling the apneic patient; however its relevance in testing has been challenged, since there are controversies in the literature as to its capacity to detect the upper airway collapse site, of predicting the oropharyngeal surgery success and OSAS severity.

In our service, we used the nasofibroscopy to complement the neck and orofacial physical exam in patients with suspected OSAS; however the Muller's maneuver has a fundamentally didactic intent, with little input regarding the treatment approach for the apneic patient.
